# Physical, Modular and Articulated Interface for Interactive Molecular Manipulation †

**DOI:** 10.3390/s20185415

**Published:** 2020-09-21

**Authors:** Bastien Vincke, Mohamed Anis Ghaoui, Nicolas Férey, Xavier Martinez

**Affiliations:** 1 SATIE—CNRS UMR 8029, Univ. Paris-Saclay, Digiteo Labs, BAT 660, 91405 Orsay CEDEX, France; mohamed-anis.ghaoui@universite-paris-saclay.fr; 2 VENISE Team, LIMSI, CNRS, Univ. Paris-Saclay, 91405 Orsay, France; nicolas.ferey@limsi.fr; 3 Laboratoire de Biochimie Théorique, CNRS, UPR9080, Univ. Paris Diderot, Sorbonne Paris Cité, 75013 Paris, France; martinez@ibpc.fr; 4 Institut de Biologie Physico-Chimique-Fondation Edmond de Rotschild, PSL Research University, 75006 Paris, France

**Keywords:** virtual reality, tangible interface, human–machine interface, molecular simulation, internet of things, rational drug design

## Abstract

Rational drug design is an approach based on detailed knowledge of molecular interactions and dynamic of bio-molecules. This approach involves designing new digital and interactive tools including classical desktop interaction devices as well as advanced ones such as haptic arms or virtual reality devices. These approaches however struggle to deal with flexibility of bio-molecules by simultaneously steering the numerous degrees of freedom. We propose a new method that follows a direct interaction approach by implementing an innovative methodology benefiting from a physical, modular and articulated molecular interface augmented by wireless embedded sensors. The goal is to create, design and steer its in silico twin virtual model and better interact with dynamic molecular models.

## 1. Introduction

Rational drug design is based on a detailed understanding of both protein-ligand interactions, and protein functions. Each protein activity depends on many factors: its three-dimensional structure is mainly driven by its physico-chemical properties, dynamic properties and bio-mechanical behaviour, and its interactions with other partners. This information and, particularly, the dynamics of these molecules are difficult to obtain through experimentation. This is why numerical simulation tools have been designed to obtain theoretical results, models that have to be validated by experimental data. Nowadays, molecular simulations tools are highly performant and offer to visualize and interact with the simulated molecules. This opens new perspectives to build, simulate and test models and hypotheses in interactive time to eventually understand and design new drugs.

However, no specific tool has been designed to interact with these complex, flexible and dynamic molecular objects to overcome the major hurdle of this approach.

Human–machine interfaces for molecular simulations began in the 1970s. At that time, haptic interfaces [1] were excessively expensive and voluminous. Nowadays, one can find haptic devices at low cost and they are common in offices or virtual reality environments. Nevertheless, interacting with molecular simulations using haptic arms can be limited, especially for highly dynamic experiments like molecular dynamic simulations. Indeed, flexible proteins are complex objects rotating and translating on a large set of axes, larger than those that offer six degrees of freedom of most 3D interaction devices  [2]. An initial solution was provided by Arthur Olson’s team to transform physical models into more tangible and richer interfaces than classical interaction devices (keyboard, mouse). Tangible interfaces combine physical and virtual molecular representations, thanks to augmented reality techniques while easing manipulation of the molecular model. The approach was based on an explicit marking system (ARToolkit) or retro-reflective infrared markers, leading to occasional tracking loss due to marker occlusions during manipulation.

**Figure 1:**
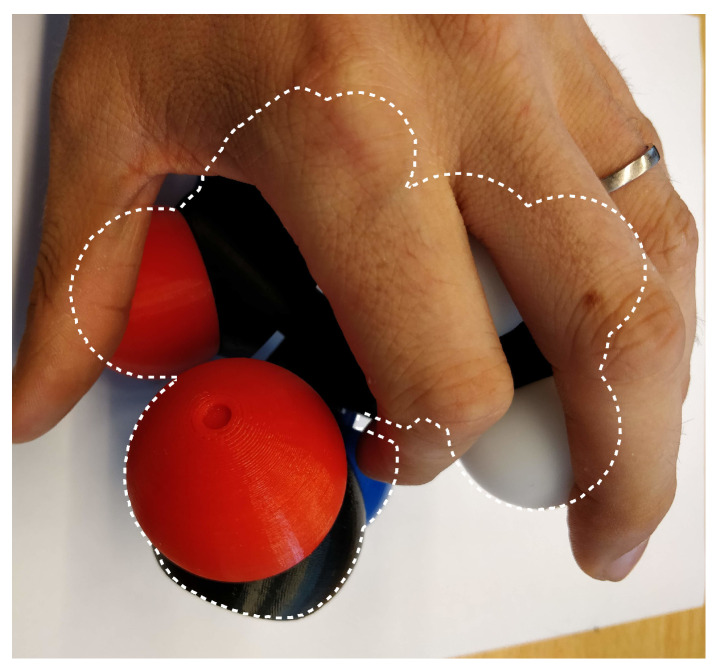
Occlusion problem with the usage of image tracking.

Using markers for this task is often problematic, thus, Martinez et al. proposed a robust, non-intrusive and marker-free approach to reconstruct a three-dimensional digital molecular model from the modular and flexible physical object manipulated in interactive time  [3]. The approach is based on a single camera leading to a major occlusion problem (Figure 1).

Jacobson proposed a generic tangible interface to control articulated virtual characters [4]. Jacobson’s team shows the usage of this humanoid structure into animation applications, to control a virtual avatar by binding its elemental structures to the physical model. Glauser also created a deformation capture technique using a foldable sensors array with a customized sensors placement and training its neural network with a dataset [5]. Even if these interfaces show promising results, they both use wired connectivity, do not provide automatic pairing, and thus, are not suitable for common protein manipulation.

Wireless interfaces, especially for bio-electronics  [6,7], are still in development. Even if the solution is not wired, it often remains non-modular and non-portable. Once implemented, the interface is usually not designed to be connected/reconnected quickly and easily.

To improve interacting with molecular simulations, we need a physical structure that can represent each protein using different parts that can be easily connected to each others. This model has to be flexible to replicate the high and important protein flexibility and wireless to increase its modularity and usability.

We previously presented an initial version of our interface  [8]. Here, we introduce many details concerning the management of the molecule network as well as the man–machine interaction and the simulator we have developed to visualize the reconstructed molecules.

## 2. Contribution

**Figure 2:**
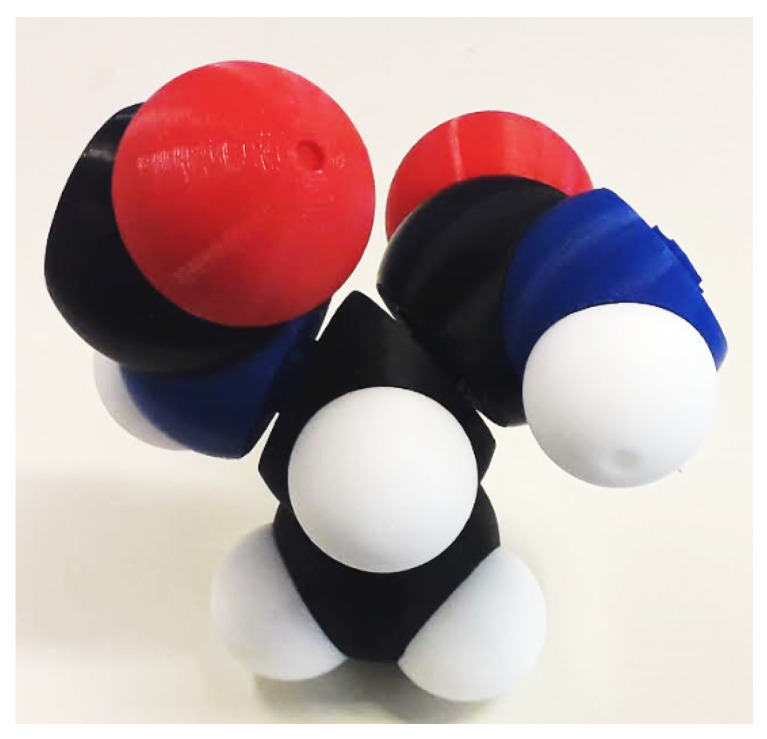
New assembled colour-coded model.

**Figure 3:**
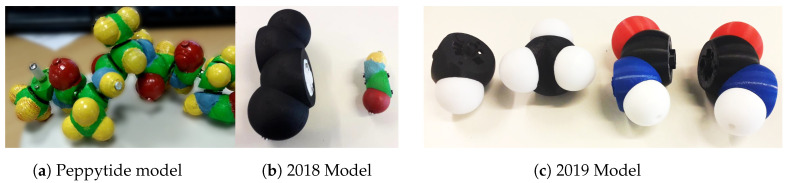
Different physical models for PeppyTide.

We designed an instrumented tangible molecular interface (Figure 2), allowing to drive a digital molecular avatar via a modular and flexible physical model. Our interface is based on the Peppytide molecular model (Figure 3a). Peppytide is a coarse-grained molecular model  [9], currently composed of 3D-printed modules that can be assembled to form a rigid body chain. We partly redesigned the model to integrate necessary electronic parts to measure the chain’s topology. Our interface allows us to both reconstruct and manipulate accurately a virtual molecule by driving a large set of degrees of freedom.

## 3. Materials and Methods

Several modifications were made to the model to allow the integration of electronic parts. We modified the structure of the model to accommodate an electronic board equipped with an angle sensor, a battery and a wireless communication system. In this section, we present these modifications to ease the replication of this interface.

### 3.1. Mechanical Structure

The integration of the electronics cannot be done without accessing the inner part of atoms and having enough space to accommodate an electronic board. Firstly, we had to double the dimensions of the Peppytide model. We transitioned from a scale of 1 Å: 9.35 mm to a scale of 1 Å: 18.7 mm.

The model is now twice as big as the original one with each atom being hallow.

**Figure 4:**
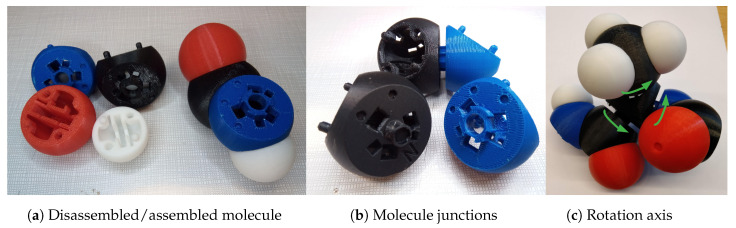
Current physical model mechanics.

Then, we developed a system of assembly for 3D printed atoms. On each atom, there are two shafts and two holes. The assembly is performed by simply encasing the two pieces. This method allows both the placement of electronic components inside atoms and printing the atoms in conventional colors making them easily distinguishable by users. Now that the atoms can be attached to each other we can assemble a molecule part, as shown in Figure 4a.

Each part needs to be connected to another according to the biological model, still allowing to rotate around the connection axis. Thus, we designed a hole and shaft connection mechanism where the shaft plugged in the hollow tube constrains the molecule to rotate around the junction axis. The tip of the shaft has an indentation that acts as a socket for a magnet (used for the angular sensor) to be placed in. A rotation axis example is given in Figure 4c. On the plinths of these junctions, we kept the magnets of the Peppytide model to simulate potential peaks and favorable molecule positions.

Finally, on the electronic board, many components have to be visible from the outside. We must add sockets, see in Figure 4b, at the pivot junctions to allow the IR LED and the photo-diodes, presented in Section 3.2.5, for on-sight communication.

### 3.2. Electronics Hardware

**Figure 5:**
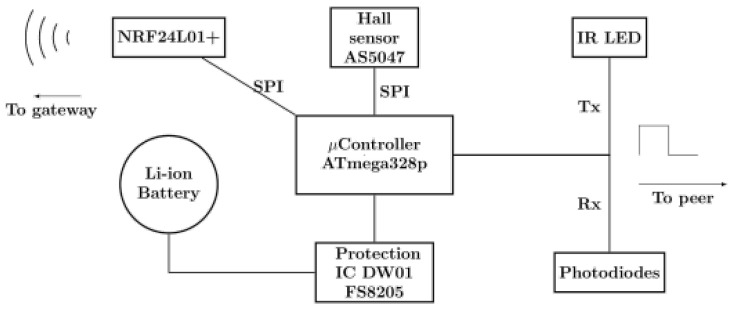
Block diagram: Embedded electronics.

Each of the previously mentioned pivot junction may be equipped with the following set of electronic devices (Figure 5) to “bring it to life”, allowing it to be interactive and connected to a gateway μcontroller. The following block diagram summarises all the embedded electronics:

- *ATmega328p*μC [10].
- *NRF24L01*+ [11].
- IR LED.
- 3 Photodiodes.
- Magnet rotary position sensor.
- Protection IC.
- Lithium-Ion Battery.

It is important to note that it is not necessary to have all of these components on every electronic board. Some components may be discarded in order to suit the needs of the current molecule part and to match the constraints of the Peppytide model and molecule specifications presented in Section 3.3.

#### 3.2.1. Micro-Controller

**Figure 6:**
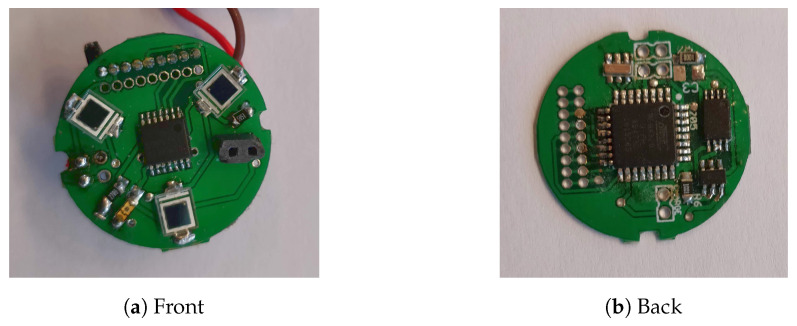
Electronic board implemented at each junction.

The main chip is an *ATmega328p* μcontroller that interfaces its surrounding modules either by SPI Bus or digital pins (Figure 6). This μcontroller is cheap, low power, robust and widely supported by the Arduino community with all the necessary libraries. Thus, it is the most suitable choice for these prototypes.

#### 3.2.2. Angular Measurements at Joints

Angle measurement is performed using an *AS5047P* hall effect sensor visible in the middle of Figure 6a. This kind of sensor provides a very accurate (14 bits) and non-contact angle measurement. The axis is equipped with a diametrically polarised magnet. The sensor is placed in front of this axis and is interfaced using an SPI bus. The sensor delivers two important data: the angle value and the magnetic field value. The former will serve to reconstruct the molecule based on the backbone angles, while the latter will detect if another part is plugged in or not.

#### 3.2.3. Battery

The interface is equipped with a Lithium-Ion 3.6 V 150 mAh CR2450 battery. This battery is compact, light and rechargeable. Each molecule has its own battery. If a molecule contains more than one junction, we use the same battery for all the junctions. Lithium-Ion batteries must be protected against deep discharge. Therefore we have added a protection circuit with two components (FS8205S, DW01) to protect the battery. For recharging, each molecule can be connected to a charger. The autonomy of the model depends on the number of junctions connected to the battery. However, we observed that the interface consumes around 40 mA with two junctions. The autonomy is therefore about 3 hours of use, which suits our specifications.

#### 3.2.4. Wireless Interface

**Figure 7:**
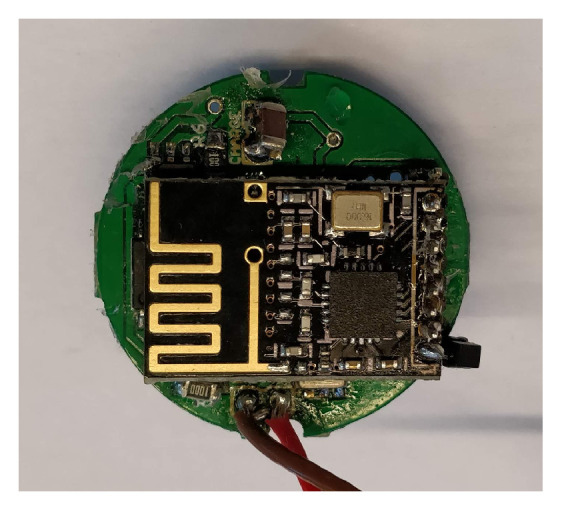
Board with Routing Facility (RF) module.

The virtual reconstruction of a protein chain requires the deployment of a communication network between each molecule and the base gateway that is connected to the simulator. The NRF24L01+ [11] is the Routing Facility (RF) module that allows this because it is a low power, cheap and efficient wireless chip that allows the construction of a wireless network  (Figure 7).

##### NRF24L01+

This module communicates in the ISM band (2400–2500 MHz) [12] over 126 channels spaced by a minimum of 1 MHz [11]. It also offers:

Amplification levels:

- RF24_PA_MIN = −18 dBm
- RF24_PA_LOW = −12 dBm
- RF24_PA_HIGH = −6 dBm
- RF24_PA_MAX = 0 dBm

Communication rate:

- RF24_250KBPS
- RF24_1MBPS
- RF24_2MBPS

The performance and energy consumption of the module depends on these two parameters. In Tx, at 0 dBm, the module drains up to 11.5 mA. While in Rx, at 2 Mpbs, the module drains up to 13 mA. So, at its highest performances, the *NRF24L01+* is more energy-efficient than other comparable modules like WiFi ESP or Bluetooth.

For this project, we set RF24_PA_MIN = −18 dBm power amplification and RF24_250KBPS communication rate, unless specified otherwise.

##### Duplex Communication

**Figure 8:**
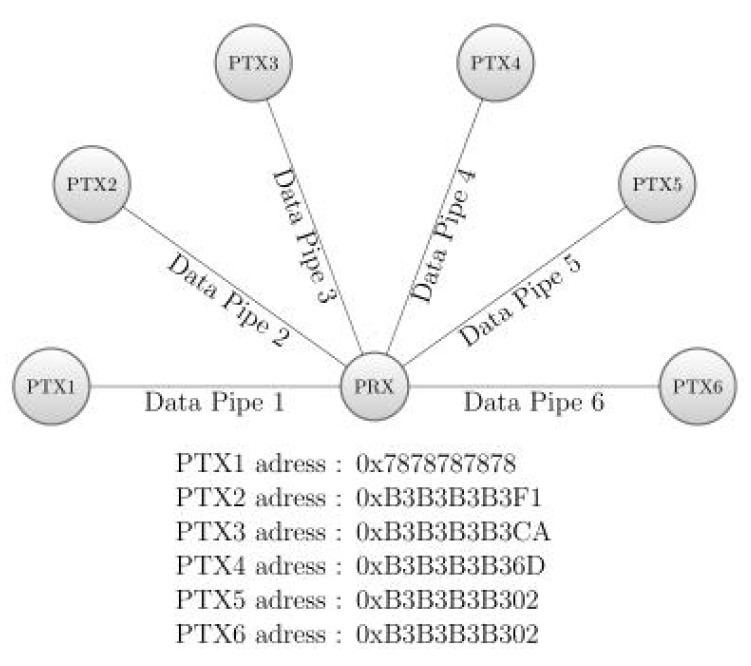
Seven modules connection scheme.

Even though this module can only perform duplex communication, its communication rate is more than enough to make it seem full-duplex and to allow interactivity between the simulator and the end-user. To begin with, we need to establish an Rx ↔ Tx communication between two modules by interfacing each with the RF24 library  [13]. To communicate, each module has to open a “pipe” to read/write on a specific address and to cross write/read from the other μController. Then, we need to build a network based on this simple principle. However, the NRF24L01+ can only connect to six other modules [14] as shown on Figure 8. Thus a tree topology must be built in order to accommodate more nodes in the network.

##### Static Network Topology

To facilitate the setup of such a tree, the use of the NRF24Network library [15] is convenient. This sets up a tree with each node having five children and each node communicates by knowing the target address. This scheme has minimal latency and provides up to 3125 ($5^{5}$) modules in a network (a frequency channel). Although this topology is performant, if one of the nodes happened to get disconnected for any reason, all its children nodes will be disconnected and that part of the tree collapses. So, we need to give the node the possibility to reroute and always be able to reach the gateway.

##### Dynamic Network Topology

**Figure 9:**
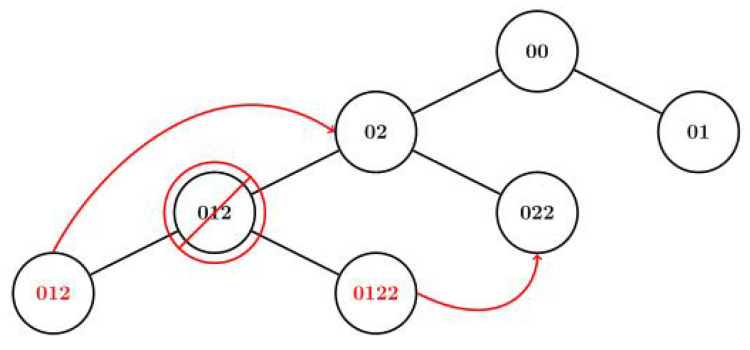
Dynamic tree rerouting.

This topology allows the nodes to reconnect even if their parent has disconnected making them immune to “sudden death” as shown in Figure 9. Each node has a unique ID written in its EEPROM to store it even if the power supply of the node is disconnected or if it goes out of range.

The routing is performed by the gateway which has 0 as its ID and a routing table written in its EEPROM. In this case, the addresses are completely transparent, only the ID is required to communicate and also allows to use a diffusion ID to all nodes. Such a dynamic network is supported by the MySensors library  [16]. Although this change of topology makes the network immune to sudden death and introduces minimal latency, it reduces the number of total possible nodes to 254 (excluding 0 and 255). Because the ID is an 8 bits unsigned value and some of the previously mentioned pipes are used for routing purposes. In this case, if a larger network of molecules is desired, we can simply connect more gateways, each on a different frequency channel. Here we also propose few strategies to use the available IDs more efficiently in Section 3.3.

#### 3.2.5. Automated Molecular Pairing

**Figure 10:**
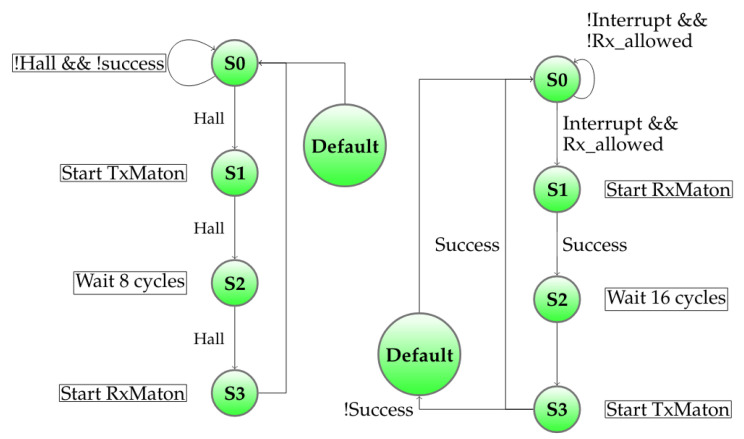
Automatic pairing automatons: left is sensor-maton, right is magnet-maton.

When two molecule parts are plugged together, one has a magnet, the other has the previously mentioned hall effect sensor. As soon as the master sensor measures a magnetic field above the experimental threshold (4000 units), it starts an asynchronous timer-interrupt based communication protocol that the slave magnet was waiting for. The automatons for each of the master and slave are presented in the Figure 10.

**Figure 11:**
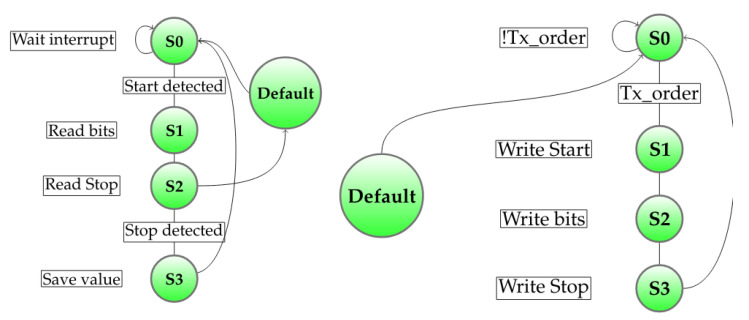
Rx (**left**) and Tx (**right**) automatons.

Each of the automatons uses two other generic automatons: Rx-maton and Tx-maton. These are responsible for reading Rx and writing Tx when allowed to. These automatons, which are shown in Figure 11, operate on 16 bits Timer1 overflow interrupt to read/write Rx/Tx. Note that the Rx-maton is waiting for its Rx pin interrupt to trigger.

So only the Master sensor and Slave magnet can give the order to communicate or not. Since we want a nearly instantaneous pairing time, we need the bit rate to be at least 1000 bps which means send a bit every 1 ms. By splitting a bit into eight intervals, we allow the sequencing of special symbols like START and STOP. Thus, timer 1 is configured to an interrupt overflow period of 125 μs.

##### IR Communication Device

**Figure 12:**
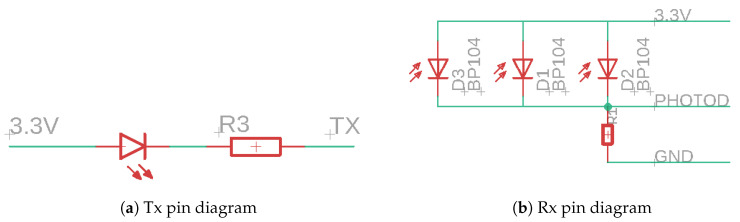
Asynchronous communication electrical diagrams.

Now that the automatons are ready to operate, they need a means of communication, a device. We chose to set up one IR LED and three photo-diodes. The former is placed in a PULL_UP mode as displayed in Figure 12a and to correctly write on Tx, we need a NOT gate on the output pin. So the Tx pin uses an inverted logic.

For Rx, all three photo-diodes are connected in parallel as shown in Figure 12b, meaning that if one of them receives IR electromagnetic energy, its voltage drop to 0 and thus the Rx pin is connected to 3.3 V. So the Rx pin uses a direct logic.

##### Exchanged Data

**Figure 13:**
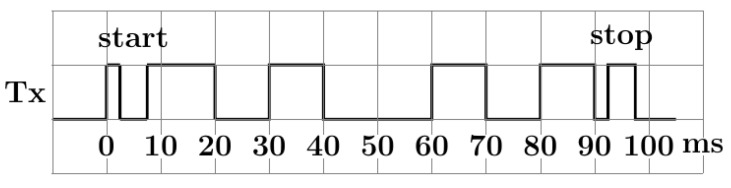
Tx line visualised: sending 0xA5 (LSB to MSB).

When the Master hall try to pair with its neighbor, it will send its 8 bits ID though Tx, as shown in the chronograph Figure 13, and waits for 8 bits neighbor ID and 8 bits check ID. If the check ID is the same as its own ID, then the pairing was successful, otherwise the pairing restarts. There is a total of 32 bits exchanged within a period of 1 ms per bit:

$$T_{pairing}=T\left(\mathrm{START}+8\;\mathrm{bits}+\mathrm{STOP}+4\;\mathrm{dead}\;\mathrm{bits}+\mathrm{START}+16\;\mathrm{bits}+STOP\right)=32\;\mathrm{ms}$$

There are two dead bits (16 cycles) where the master Tx has to wait to ensure that slave Rx is ready to receive. When the pairing is successful, it is allowed to interact with the gateway as presented in Section 3.3.

#### 3.2.6. Over the Air Programming

During the preparation of some prototypes, one difficulty was that the reprogramming of the μ-controller inside the module was not possible without tearing the part to pieces. Thus an OTA-enabled [17] μ-controller was needed.

##### Bootloader

The bootloader is a start-up program that is called with each reset of the ATMega328p chip before the main program. The default bootloader for this chip provided by Arduino [18] is set up to listen to the serial port for 4 s and intercept the incoming bytes, if there is any, it locks itself into update mode. The intercepted byte-stream consists of a compiled code for the new main program to be overwritten in the flash memory and then executed.

To perform an OTA update, we use the MySensors bootloader [17,19] which changes the default bootloader and adds the interception of the incoming bytes from the Network Routing Facility (NRF). If any data was available, the bootloader locks itself in update mode. It can retrieve the updated program by requesting it from the gateway. The data coherence is ensured by the bootloader requesting the new firmware page by page, 16 bytes per page and will act in burst mode trying to get as much page as possible in succession. The watchdog forces a page request every 4 s ensuring a minimal update speed of 16 bytes/4 s and preventing any possible bricking. Once the update finished, the bootloader releases the chip and allows the main program to start.

#### 3.2.7. Base Gateway

This component is simply an *ATmega328p* with an NRF and USB connection. It has three major features: sending serial data to the simulator, communicating and routing each node in the network, delivering updates to each node requesting it. It only has to be plugged-in in order to perform its duty.

### 3.3. Structure Specifications

In this part, we explain what strategies were adopted to optimize the limited resource usage and reduce the bandwidth overloading.

#### Passive Molecule

While planning for a building strategy, we noticed that some type of molecule parts was more complicated than others. The complexity varies in size, shape, plugging possibilities and simulator implementation aspects. For instance, the Methyl is the simplest molecule part to build, assemble and simulate because it only has once junction, or face, to be connected with and can only rotate around that axis. Meanwhile, the α-Carbon, has three connectable faces and can have a rotation on any of them meaning that a single carbon atom has to accommodate three electronic boards with each a NRF and a Hall sensor. Firstly, it is costly to have twice the same NRF chip. Secondly, the size of the carbon atom doesn’t allow for such a placement. Finally, this adds complexity to the programming side in the simulator. So in order to reduce this complexity, the α-Carbon has been reduced to a passive molecule part with only three electronic boards, three magnets, three IR communication devices, and a single battery. This gets rid of the NRF and hall sensor. This also means that, out of the NRF unique IDs distribution shown in Table 1a, α-carbons can be relocated to IDs that are beyond 255, since they do not require an NRF at all.

### 3.4. Peppytide Unity Simulator—PUS

The implementation of the rendering and the simulation is done in Unity3D that provides both a high-performance rendering pipeline combined with a reliable physic engine (Nvidia PhysX). In order to construct, display and articulate the protein avatar correctly, we need to instantiate virtual molecular objects.

#### 3.4.1. Conventions

Every dimension/measurement is giving in either mm or ${}^{\circ }$ unless specified otherwise.

Because Unity uses the left-hand convention, the axis and their positive and negative directions have the following name:

- Positive X-axis: Right
- Negative X-axis: Left
- Positive Y-axis: Up
- Negative Y-axis: Down
- Positive Z-axis: Forward
- Negative Z-axis: Back

x=ρcos(θ)sin(φ)

y=ρcos(φ)

z=ρsin(θ)sin(φ),

where θ is the angle from X-axis to Z-axis and φ is the angle from Y-axis to Z-axis, unless specified otherwise.

In this document, we use conventional atomic symbols and colors for Carbon (C), Oxygen (O), Nitrogen (N) and Hydrogen(H). Some programming symbols may indicates that atoms belong to a special entity. We define them as:

- **Cr**: Carbon radical—Methyl’s Carbon.
- **Ca**: Carbon alpha (The adj + noun English rule has been inverted for convenience)—α-Carbon’s Carbon.
- **C**: Carbon—Amide’s Carbon.

#### 3.4.2. Molecule Classes in Unity

**Figure 14:**
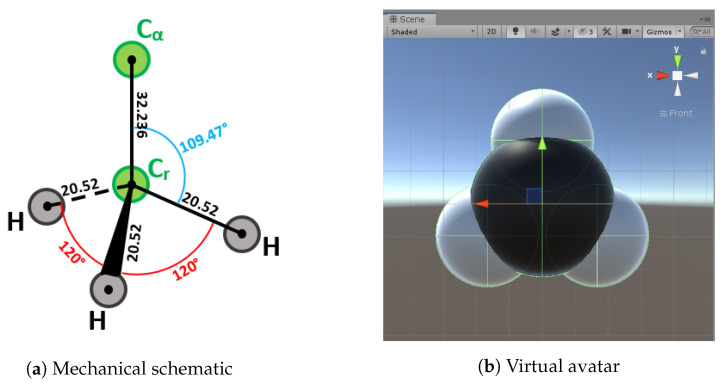
Physical and virtual methyl representations.

**Figure 15:**
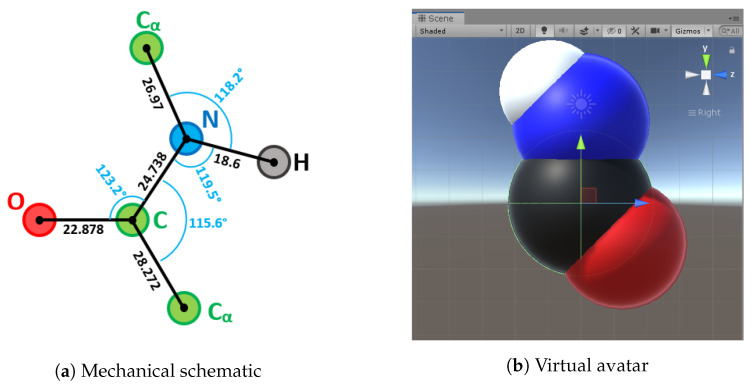
Amide part representations.

**Figure 16:**
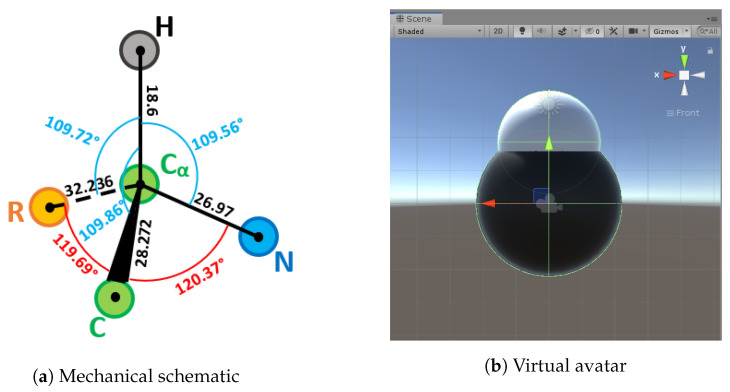
Alpha carbon representations.

The geometric references of the elementary molecule parts, which are Methyl, α-Carbon and Amide, are chosen in a way that facilitates their relative placement and interactions (Figure 14, Figure 15 and Figure 16). We tried to find as many common geometric features as possible (symmetry, co-linearity, axis invariance...) to build each class. Every atom object in the scene is a sphere mesh, set to a certain color. These spheres are given a parent (see Section 3.4.4) and are placed in their corresponding relative positions.

##### Methyl, C and N Term

The pivot junction is on the forward axis which implies that, once this type of molecule is plugged to an α-Carbon, it can only rotate around its own Z-axis.

#### 3.4.3. Connections

The connections are made to facilitate the placement of each molecule relatively to its parents molecule. This means maximising the use of common geometric features to minimise computation complexity. Based on Promita Chakraborty’s Peppytide model [9]:

- Methyl, C and N Term can only connect to an α-Carbon.
- An amide can only be connected to an α-Carbon, either by its C or N face.
- An α-Carbon is unable to rotate on its own.

#### 3.4.4. Linking and Parenting

Any transformations applied to the parent of a molecule part is reflected on its children. This makes building the protein chain a lot easier as linking correctly every molecule part to each other is the only step needed.

#### 3.4.5. Chain Building

Now that the molecules objects can be dynamically instantiated, they have to be set in a certain data structure to store them. A dictionary associates the unique ID of each molecule part as key, to the instantiated molecule part as a value.

### 3.5. Disconnections

When a molecule of the physical model is unplugged, its avatar and all linked parts have to be destroyed/hidden from the scene. To perform this, we can destroy every atom of the targeted ID molecule and then just remove it from the dictionary container. If a re-connection is needed, each class contains pair-face ID of its neighbour molecules and can recursively rebuild the whole sub-chain. This offers the possibility to turn PeppyTide Unity Simulator (PUS) into a multi-protein manager, building many sub-chains and connect all of them to have a large amino-acid chain. This feature is mandatory because most of users tend to build several sub-chains when connect them in the end.

### 3.6. Interactions

The main goal of this project was simplifying the human–machine interaction with the simulator through an instrumented mechanical interface. While that is convenient and is already easier to use than the standard mouse-keyboard device, there are possibilities to use one mode or combine many them together.

#### 3.6.1. Specialised UI Interaction

One could construct an User Interface (UI) system to construct and control each molecule part of a peptide chain. For example, one could create a button to add an α-Carbon part and another button to link it to the selected part on a link that is asked to the user by clicking on it. One could also provide sliders to modify angles between each part.

Combined with classical mouse and keyboard interaction methods, this could be an efficient way to interact with the virtual model.

#### 3.6.2. Command-Line Input

**Figure 17:**
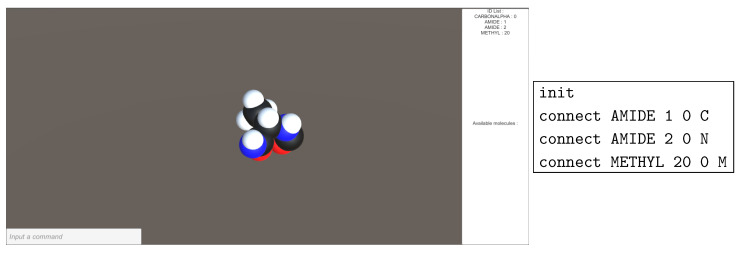
Command Line Input (CLI) generated molecules.

This mode is intended to interact with PUS using the command-Line to build and modify a peptide chain. This will us allow to create and link molecule parts without necessarily holding the model in hands. This mode also compensates for the lack of physical molecules and helps the creation of several replicas of protein chains. Figure 17 gives an example of molecules instantiated with this mode. The command is parsed, validated with asserted inputs and then executed.

This mode also offers the possibility to save and load molecules that were built with the Command Line Input (CLI) into .pusmol files by recording every valid command that was entered.

#### 3.6.3. Hard Coded Implementation

**Figure 18:**
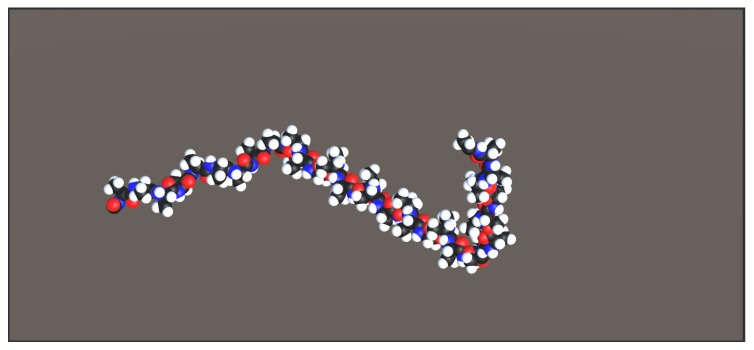
Code generated protein chains.

Hard Coded Implementation (HDI) is the least user-friendly tool and should be used only for procedural generation, demonstration and benchmarking. It is up to the programmer-user to verify the coherence between their code and the API. The same previous example can be instantiated with the following C# script: 
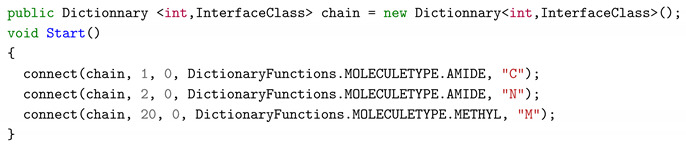


This mode is not recommended for users who do not have programming capabilities in C#, object-oriented or/and Unity3D because it requires the implementation of a script in the scene of the game and instantiate the chain in a procedural manner. The chain shown in Figure 18 shows two generated animated chains thanks to a script in C#. The main interest of this method is to be able to create very large molecules. The created molecules will not be made by hand but described via scripts or loops.

#### 3.6.4. Physical Model Interaction

**Figure 19:**
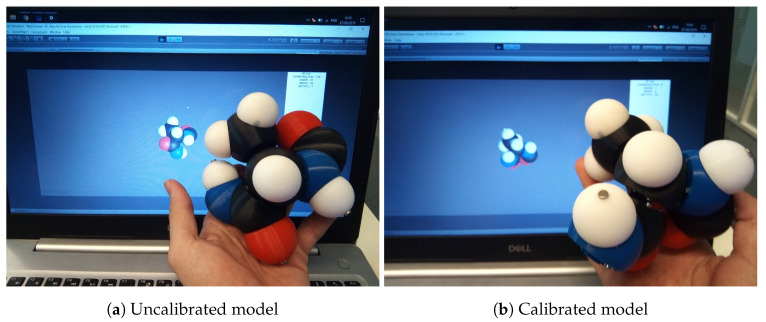
Offset representations in the simulator.

Using the physical model as a tangible interface is a direct interaction, and it seems to be the most efficient for users because it benefits from the tangible model accuracy to control the avatar in Unity3D. Figure 19 gives an example of this mode where the user only has to connect/disconnect some molecules to others and build a chain.

Constructing and deconstructing amino-acids using this tangible interface is also straightforward because of the 3D nature of the interaction and human facilities to manipulate tangible objects. It also teaches users biochemical information about proteins.

### 3.7. Magnet’s Offset Correction

**Figure 20:**
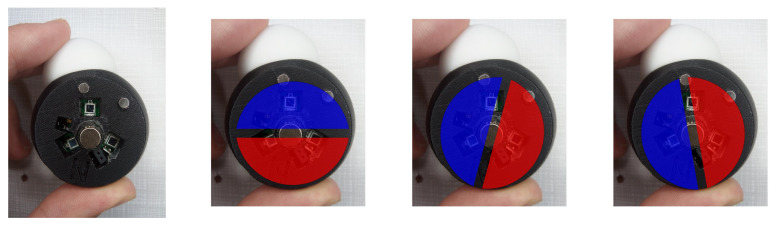
Unknown offset representations on an Alpha Carbon magnet.

One problem we faced during the assembling of model parts was that the radially polarised magnets shown in Figure 20, once glued to the tip of the shaft, had different orientations. So each of them has an offset that needed to be corrected.

Thus, we need to associate an angle value to each electronic board that has a magnet on it. Since each of them is identified with an unique ID, we simply implement a static constant table containing these values, measured experimentally by taking one magnet as reference. Once these magnet offsets are calibrated, the virtual model behaves accordingly to the physical model as shown in Figure 19.

It is important to note that the Hall sensors used to measure magnetic fields between every junction have a very negligible offset ($\pm 1^{\circ }$) thanks to the mechanical structure that compels us to place the electronic board into its dedicated slot, leaving a placement error due to soldering and prototyping hand manipulations.

### 3.8. First Evaluation

We conducted a first batch of experiments to highlight the perks of each mode of interaction. Several conclusions emerged very quickly from the first evaluations.

Firstly, it seems to be very difficult to correctly construct a molecule using Hard Coded Implementation without visual feedback. Users absolutely need visual feedback to build the molecule. Contrary to what we thought, the command line interaction mode is relatively simple to use for a simple model. Users can build a model after reading a simple instruction manual and build a molecule containing five modules in less than 10 min (simulator handling included).

Our interface has been very well received by users. It is a mode of interaction that very few users are familiar with. Therefore, it is a novelty that attracts the user to try this technology. The use of the interface is very simple, there is no major difficulty in building a molecule.

However, the main issue we noticed is the difficulty of visualizing the molecular avatar due to the lack of global orientation of the interface. Indeed, our interface only measures the relative orientation of an atom compared to another. It does not measure the global orientation of the interface. Users can, therefore, rotate the interface on itself without the simulator reacting. This is the main feedback from users about our interface. It, therefore, seems essential to measure the overall orientation of the interface and to take it into account with the simulation.

Tracking the global orientation of the interface could be done in several ways. First, we could add several cameras in the environment and try to find the orientation of the molecule in the images. However, this process will always suffer from occlusion problems due to the manipulation of the interface. The second possibility is to use an Inertial Measurement Unit in one or more atoms. This solution seemed promising to us but our interface embeds several magnets and this strongly disturbs the magnetometers of our IMU. Only accelerometers and gyroscopes can be used. It is thus possible to measure the rotations as well as the orientation of two axes but we cannot measure the complete altitude of the interface.

Following the first batch of experimentation, it also appeared that it will be important to miniaturize the interface. Indeed, it is quite usable today but if we want to obtain a large polypeptide chain, it will be essential to reduce the size of each atom.

## 4. Conclusions

In this work, we proposed an instrumented tangible molecular interface allowing us to drive a digital molecular avatar via a modular and flexible physical model. The proposed interface is wireless, autonomous and communicates with a gateway allowing a bidirectional exchange with the computer in charge of the simulation. The interface allows us to control quickly a very large number of degrees of freedom. The aim of our user-centered approach was to better consider expert knowledge, while addressing the important combinatorial issues inherent in high throughput screening approaches. Moreover, it is easy to use and does not require any special skill or training.

We are currently experiencing several use cases with domain experts. The first one is to build and manipulate a complete small peptide for research and pedagogical purposes. This is a use case in the Peppytide passive model designed by P. Chakraborty, who experimented with longer chains (around 20 amino acids) folding of several secondary structures. Note that the model was laid on a table and partly manipulated. In a second use case, our molecular interface is linked to a selected component to manipulate subparts of the full molecule displayed in its digital form. In the short term, we also plan to apply our approach to design other kinds of bio-molecules like drugs with comparable size. The goal is to interactively modify flexible drugs while having simulation feedback.

A quantitative study will be conducted to compare the performance of our interface compared to a more traditional method using a mouse or command lines as an interface.

## Figures and Tables

**Table 1:** Molecule ID distribution.

Molecule	ID		Molecule	ID
Methyl	1–40		Methyl	1–85
Amide	41–80	⟹	Amide	85–255
Carbon Alpha	81–120		Carbon Alpha	257–510
(**a**) Current distribution			(**b**) Possible better distribution	

## Abbreviations

The following abbreviations are used in this manuscript:

CLI	Command Line Input
HCI	Hard-Coded Implementation
IR	Infra-Red
NRF	Network Routing Facility
OOP	Object-Oriented Programming
OTAP	Over The Air Programming
PUS	PeppyTide Unity Simulator
